# Baseline thyroid indices and the subsequent response to citalopram treatment, a pilot study

**DOI:** 10.1002/brb3.109

**Published:** 2013-01-18

**Authors:** Osama A Abulseoud, Michael Gitlin, Lori Altshuler, Mark A Frye

**Affiliations:** 1 Department of Psychiatry and Biobehavioral Science, David Geffen School of Medicine at UCLALos Angeles, CA; 2 Department of Psychiatry and Psychology, Mayo ClinicRochester, MN

**Keywords:** Acceleration, antidepressant response, citalopram, free thyroxine, thyroid, thyroid-stimulating hormone, triiodothyronine

## Abstract

The lack of reliable outcome predictors and the delayed onset of therapeutic response to antidepressants are among the clinical challenges in the treatment of depression. Identifying clinical correlates associated with antidepressant response would reduce symptom severity and morbidity for patients with depression. Twenty-three subjects with major depression were treated with citalopram 20 mg/day in a 6-week open trial and were also simultaneously randomized to either adjunctive triiodothyronine (T3) 25 *μ*g BID (*n* = 7), pindolol 5 mg BID (*n* = 8), or placebo (*n* = 8). Baseline thyroid-stimulating hormone (TSH), FT4, FT3, and TT3 were measured for potential relationships to treatment response across groups. In males only, there was a significant inverse correlation between baseline free T4 and time to response (*r* = −0.7, *P* = 0.034). In both males and females across all treatment conditions, as measured by Kaplan–Meier (K–M) maintenance failure time, baseline TSH below the mean (1.5 ng/dL) was associated with a shorter time to response (50% reduction in Montgomery and Asberg Depression Rating Scale [MADRS] score) (χ^2^ = 4.53, df = 1, *P* = 0.03). Patients with baseline TSH above the mean were less likely to reach full remission (MADRS ≤ 7) (χ^2^ = 4.38, df = 1, *P* = 0.03). No significant differences between groups emerged in the mean response time. Baseline thyroid function, as measured by serum free T4 and TSH, may predict a patient's response time to antidepressant treatment with citalopram.

## Introduction

Major depression is a serious medical illness that affects more than 13% of adults in the U.S. during their lifetime (Hasin et al. 2005). The vast majority of depressed patients have normal peripheral thyroid indices (Joffe and Levitt 1993). However, the prevalence of subclinical hypothyroidism is significantly higher among depressed patients compared with the general population (Haggerty and Prange 1995) and over 63% of patients with subclinical hypothyroidism report depressive symptoms (Demartini et al. 2010). A rich body of literature has focused on thyroid hormone indices as predictors of antidepressant response outcome. In one study, relatively elevated free T4 index in depressed men was associated with a faster antidepressant response time as measured by length of hospital stay (Abulseoud et al. 2007). Lower pretreatment thyroid-stimulating hormone (TSH), within normal range, was found to predict better response to antidepressant treatment in some studies (Amsterdam et al. 1996; Berlin and Corruble 2002; Gitlin et al. 2004), but not all (Joffe and Singer 1987; Fava et al. 1995; Sokolov et al. 1996; Szadoczky et al. 2004; Gambi et al. 2005; Brouwer et al. 2006).

Another clinical challenge stems from the fact that less than half of treatment-seeking depressed patients reach the expected therapeutic benefits after several weeks of adequate antidepressant treatment (Blier and de Montigny 1994; Warden et al. 2007). This delayed onset of response triggered the search for accelerating agents such as triiodothyronine (T3), first suggested by Arthur Prange and collaborators over four decades ago (Prange et al. 1969). There is some evidence that adding thyroid hormone at the onset of initiating antidepressant treatment could shorten the delay of antidepressant effect. In our meta-analysis (Altshuler et al. 2001) of six randomized double-blind controlled studies evaluating the efficacy of T3 in accelerating the antidepressant effects of TCAs, T3 was significantly more effective as an accelerating agent compared with placebo (*P* = 0.002). The response was greater in women than men. Along the same lines, Frye et al. (1999) reported gender difference in CSF TRH in patients with refractory depression (females: 2.95 pg/mL vs. males: 3.98 pg/mL; *P* < 0.05). These gender differences at baseline; or during acceleration or treatment responses have not been prospectively confirmed in larger studies.

Another accelerating agent is pindolol, a β-blocker with activity at the 5-HT1A receptor (Blier and Bergeron 1998), which has been found in most (Pérez et al. 1997; Bordet et al. 1998; Smeraldi et al. 1998, Tome et al. 1998; Zanardi et al. 1998), but not all (Moreno et al. 1997; Maes et al. 1999) studies to shorten the time to response to selective serotonin reuptake inhibitors (SSRIs). A meta-analysis by Portella et al. (2011) found that the median survival time until first response was 65% less in the pindolol group (22 days vs. 30 days; *P* = 0.03).

The aim of this pilot study was to explore the relationship between pretreatment thyroid function measures and response to treatment in subjects enrolled in a study to compare the efficacy of T3, pindolol, and placebo in accelerating the antidepressant effect of citalopram in patients with unipolar major depressive disorder. We hypothesized that within normal range, lower baseline TSH levels will be associated with better antidepressant response outcome.

## Methods

### Subjects

All study procedures were approved by UCLA IRB. Twenty-three subjects (9 males and 14 females) with first episode unipolar major depressive disorder (DSM-IV-TR) signed an informed consent and were recruited in the study through local advertisement at UCLA campus. All 23 subjects were referred to the study either by self or by their primary care physician or psychiatrist. Diagnosis was confirmed by structured clinical interview (SCID) (First et al. 2002). Subjects with other axis I diagnosis, active suicidality, unstable medical conditions, current or past history of thyroid disease or abnormal thyroid function tests, or a positive urine toxicology screen were excluded.

### Assessments

Depressive symptoms were rated over eight visits (Screening, day 3, weeks 1, 2, 3, 4, 5, and 6) using the following rating scales: Montgomery and Asberg Depression Rating Scale (MADRS) (Montgomery and Asberg 1979), Beck Depression Inventory (BDI) (Beck et al. 1961), Clinical Global Impression – Severity of illness (CGI-S) (Guy 1976), Scale for Suicidal Ideation (SSI) (Beck et al. 1979).

### Medications

All subjects received open label citalopram (20 mg) for 6 weeks and were randomized in a blinded fashion to receive additionally triiodothyronine (T3) 25 *μ*g BID (*n* = 7), pindolol 5 mg BID (*n* = 8), or placebo (*n* = 8) at the start of citalopram treatment.

### Thyroid function tests

Serum TFTs were checked at baseline and at the end visit. TSH and total triiodothyronine (TT3) were assessed by immunoassay (ROCHE Elecsys 170 Analyzers, Roche Diagnostics, Indianapolis, IN), free triiodothyronine (FT3), and free thyroxine (FT4) by Enzyme Immunoassay Assay Diagnostic System Laboratory (EIA-DSL).

### Statistical analysis

Primary outcome measure was the time to 50% reduction in baseline MADRS scores.

Collected data were screened for distributional properties and determined to be appropriate for parametric analysis. Simple correlation analysis and proportional hazard regression (Cox Model) and accelerated failure time survival regression analysis were used to predict time to response (i.e. 50% improvement in MADRS scores) and remission (MADRS score ≤ 7) with baseline and delta TSH, FT4, FT3, and TT3 as independent variables. Analysis was done using SPSS software (SPSS IBM, Armonk, New York).

## Results

### Demographics and baseline depression scores

Of the randomized 23 subjects, 19 completed the study. Two subjects in the placebo group dropped out (one due to worsening of depression, and the other one due to excessive use of lorazepam) and two subjects missed follow-up. The mean age of the cohort was 38.34 (±12.6) years and the mean length of the index episode was 8.9 (±5.9) months with an age of onset of 32.9 (±13.5) years. All patients were required to not be receiving antidepressants for at least one month prior to starting. All, but five, patients were antidepressant naive at the time of the study. The mean baseline MADRS score was 29.7 (±5.85), BDI score was 23.4 (±7.3), and a mean CGI severity score was 4.1 (±0.3). Six patients met DSM-IV criteria of life comorbid generalized anxiety disorder, four with posttraumatic stress disorder, and one patient with social phobia. All comorbid conditions were clinically stable and none of the patients receives treatment or therapy for comorbid conditions during the study.

### The association between baseline thyroid function tests and time to response

The mean baseline TSH was 1.5 (±0.7) mIU/L, FT4 was 1.1 (±1.2) ng/dL, FT3 was 3.0 (±1.5) ng/mL, and TT3 was 120.2 (±20.9) ng/dL. No significant gender differences were observed in any of the baseline thyroid indices (Table 1).

**Table 1:** Baseline thyroid function tests in all subjects, males and females

	All subjects (*n* = 23)	Males (*n* = 9)	Females (*n* = 14)	*t*-test
TSH (mIU/L)	1.53 (±0.67)	1.27 (±0.51)	1.7 (±0.72)	*t* = 1.59, df = 21, *P* = 0.12
Free T4 (ng/dL)	1.13 (±1.17)	2.78 (±1.23)	3.65 (±1.03)	*t* = 1.83, df = 21, *P* = 0.08
Free T3 (ng/mL)	2.96 (±1.51)	2.86 (±1.05)	3.02 (±1.79)	*t* = 0.24, df = 21, *P* = 0.81
Total T3 (ng/dL)	120.21 (±20.93)	120.22 (±10.29)	120.21 (±26.0)	*t* = 0.001, df = 21, *P* = 0.99

Across genders, low baseline mean TSH was associated with shorter time to response as measured by K–M maintenance failure time (χ^2^ = 4.53, df = 1, *P* = 0.03). Furthermore, patients with baseline TSH above the mean were less likely to reach full remission (χ^2^ = 4.38, df = 1, *P* = 0.03).

**Figure 1:**
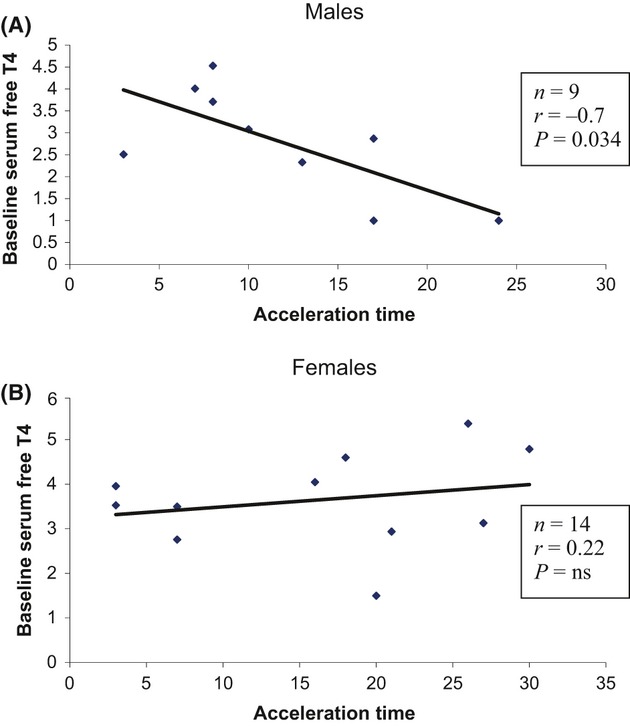
(A) Correlation between baseline free T4 and acceleration time in males. (B) Correlation between baseline free T4 and acceleration time in females.

In males only, higher baseline free T4 was inversely correlated with the time to response (*n* = 9, *r* = −0.7, *P* = 0.034 (Fig. 1A and B).

### Failure of T3 and pindolol to separate from placebo on time to reach 50% reduction in MARDS scores (i.e. response)

The mean time to response, defined as a 50% reduction in MARDS score, 14.9 (±9.1) days, was not significantly different between the three groups. T3 (*n* = 7): 16 ± 7.8, pindolol (*n* = 8): 12.2 ± 10.6, and placebo (*n* = 8): 16.4 ± 9 days. One-way ANOVA *F* (2, 21) = 0.9, *P* = 0.4. Males (*n* = 9) reached response faster than females (12.4 ± 7.6 vs. 16.8 ± 9.9 days); however, this difference was not statistically significant (*t* = 1.13, df = 21, *P* = 0.27).

## Discussion

The two major limitations of our study are (1) the open label design; and (2) the small number of subjects in each group, which may have precluded finding an accelerating effect of either medication. We estimated to require 20 patients per treatment arm based on a priori power analysis to detect a 15% difference in the primary outcome (MADRS) scores from the mean with 80% power and an alpha of 0.05. However, due to the stringent exclusion criteria (i.e. first episode, not on antidepressant, and no active comorbid axis I), the enrollment was slow and was terminated before a minimum number of intended-to-treat (ITT) subjects were enrolled. However, this pilot study with treatment group, combined allowed us to evaluate the relationship of thyroid status at baseline to treatment outcome. The study suggests that optimal thyroid function may be associated with faster response to citalopram and perhaps more so in men than in women. Low baseline TSH was associated with shorter time to response, while patients with baseline TSH above the mean were less likely to reach full remission. Moreover, higher baseline free T4 was inversely correlated with the time to response in males. We (Gitlin et al. 2004) and others (Amsterdam et al. 1996; Berlin and Corruble 2002) have reported that lower serum TSH values are associated with better responses to SSRI antidepressants and that high baseline FT4I is associated with a better antidepressant response in men as measured by a shorter length of stay in hospital for male patients (Abulseoud et al. 2007).

The significant inverse correlation between baseline free T4 and response time only in males is in agreement with our previous report (Abulseoud et al. 2007). However, the exact mechanism for this gender discrepancy is not entirely clear. Part of the mixed signals (i.e. heterogeneity) could be attributed to the use of T3 for acceleration and T4 for augmentation. T3, having a short half-life, initiated at the time of starting antidepressant treatment shortens the antidepressant response time, while T4 initiated during antidepressant treatment in refractory cases could augment antidepressant efficacy. Changes in female sex hormone during menstrual cycle could also account for some of subtle thyroid dysregulation as estrogen is known to increase the levels of thyroid-binding globulin with subsequent increase in total and decrease in free thyroxine levels (Tahboub and Arafah 2009). It could be speculated that males, compared to females, are able to activate the HPT axis and produce more thyroid hormone during a depressive episode, and T3 acceleration effect is noted more in females (Altshuler et al. 2001). However, further research is needed to better understand if the relative activation in HPT axis is pathologic or compensatory.

Perhaps due to the small sample size, coinitiating T3 or pindolol with citalopram in patients with major depression did not show a significant difference from placebo in reducing the time to response. However, Papakostas et al. (2009) reviewed five double-blind acceleration studies with T3 and found no statistically significant difference in terms of remission rates or response rates at week 1, week 2, or at endpoint between the SSRI +T3 coinitiation therapy versus SSRI monotherapy in patients with major depression.

This observation is in contrast with the significant effect of T3 in accelerating the antidepressant effect of TCAs (Altshuler et al. 2001), and the effect of pindolol in accelerating SSRIs. The median survival time until first response in Portella et al. (2011) meta-analysis was 65% less in the pindolol group (22 days vs. 30 days; *P* = 0.03). One explanation for this disparity is that T3 may shorten the response time to TCAs and not SSRIs. Of course, the small sample size of our study may have resulted in a Type II error in evaluating the accelerating response of both T3 and pindolol.

The mean time to response in our sample was only two weeks despite the relatively high baseline MADRS scores (29.7 ± 5.8). This interesting observation is in line with other published meta-analyses of double-blind randomized clinical trials reporting statistically significantly greater response to fluoxetine (Tollefson and Holman 1994) and both mirtazapine and amitriptyline (Bech 2001) compared with placebo starting from second week of treatment. This intriguing finding is difficult to fully comprehend in the face of clinical practice. Baldwin et al. (2009) and others (Stassen et al. 2007) suggested that the antidepressants differ from placebo in their ability to engender preparedness for recovery, but once triggered, recovery occurs at the same rate, regardless of the nature of the pharmacological treatment intervention.

Despite the negative acceleration results in this study, the main finding suggests that hyperactive hypothalamic pituitary thyroid axis may be associated with better antidepressant response. Further research is needed to further explore this potential marker.
